# Enhancement of antibiotic activity by efflux inhibitors against multidrug resistant *Mycobacterium tuberculosis* clinical isolates from Brazil

**DOI:** 10.3389/fmicb.2015.00330

**Published:** 2015-04-28

**Authors:** Tatiane Coelho, Diana Machado, Isabel Couto, Raquel Maschmann, Daniela Ramos, Andrea von Groll, Maria L. Rossetti, Pedro A. Silva, Miguel Viveiros

**Affiliations:** ^1^Fundação Estadual de Produção e Pesquisa em Saúde, Centro de Desenvolvimento Científico e TecnológicoPorto Alegre, Brazil; ^2^Programa de Pós-Graduação em Biologia Celular e Molecular, Centro de Biotecnologia, Universidade Federal do Rio Grande do SulPorto Alegre, Brazil; ^3^Grupo de Micobactérias, Unidade de Microbiologia Médica, Global Health and Tropical Medicine, Instituto de Higiene e Medicina Tropical, Universidade Nova de LisboaLisboa, Portugal; ^4^Faculdade de Medicina, Núcleo de Pesquisa em Microbiologia Médica, Universidade Federal do Rio GrandeRio Grande, Brazil

**Keywords:** tuberculosis, drug resistance, fluorometry, checkerboard, TEMA, fractional inhibitory concentration

## Abstract

Drug resistant tuberculosis continues to increase and new approaches for its treatment are necessary. The identification of *M. tuberculosis* clinical isolates presenting efflux as part of their resistant phenotype has a major impact in tuberculosis treatment. In this work, we used a checkerboard procedure combined with the tetrazolium microplate-based assay (TEMA) to study single combinations between antituberculosis drugs and efflux inhibitors (EIs) against multidrug resistant *M. tuberculosis* clinical isolates using the fully susceptible strain H37Rv as reference. Efflux activity was studied on a real-time basis by a fluorometric method that uses ethidium bromide as efflux substrate. Quantification of efflux pump genes mRNA transcriptional levels were performed by RT-qPCR. The fractional inhibitory concentrations (FIC) indicated synergistic activity for the interactions between isoniazid, rifampicin, amikacin, ofloxacin, and ethidium bromide plus the EIs verapamil, thioridazine and chlorpromazine. The FICs ranged from 0.25, indicating a four-fold reduction on the MICs, to 0.015, 64-fold reduction. The detection of active efflux by real-time fluorometry showed that all strains presented intrinsic efflux activity that contributes to the overall resistance which can be inhibited in the presence of the EIs. The quantification of the mRNA levels of the most important efflux pump genes on these strains shows that they are intrinsically predisposed to expel toxic compounds as the exposure to subinhibitory concentrations of antibiotics were not necessary to increase the pump mRNA levels when compared with the non-exposed counterpart. The results obtained in this study confirm that the intrinsic efflux activity contributes to the overall resistance in multidrug resistant clinical isolates of *M. tuberculosis* and that the inhibition of efflux pumps by the EIs can enhance the clinical effect of antibiotics that are their substrates.

## Introduction

Tuberculosis (TB) remains a public health issue worldwide (World Health Organization, 2014). According to the World Health Organization (2013) there were an estimated 9.0 million tuberculosis cases and 480,000 people developed multidrug-resistant tuberculosis (MDR-TB). Among these there were an estimated 210,000 deaths (World Health Organization, 2014). The TB control is severely complicated by the emergence of multi- and extensively drug resistant *Mycobacterium tuberculosis* strains. Multidrug resistant *M. tuberculosis* is recognized as *M. tuberculosis* strains resistant to at least isoniazid and rifampicin, and extensively drug resistant (XDR) *M. tuberculosis* as those resistant to isoniazid, rifampicin, a fluoroquinolone and one of the three second line injectables: amikacin, kanamycin, or capreomycin (World Health Organization, 2008). *M. tuberculosis* strains that are resistant to isoniazid and rifampicin and either a fluoroquinolone or an aminoglycoside, but not both, are colloquially termed “pre-XDR-TB” strains.

Despite the known effectiveness of the antituberculosis standard treatment against susceptible strains of *M. tuberculosis*, the first-line drugs isoniazid and rifampicin are ineffective for treating patients infected with multidrug resistant strains. Consequently, second-line drugs have to be employed. These drugs are more toxic, poorly tolerated, and sometimes difficult to obtain (Green and Garneau-Tsodikova, 2013). Furthermore, extensively drug resistant *M. tuberculosis* strains easily emerge during second-line treatment due to poor tolerance and lack of compliance (World Health Organization, 2008). The emergence and spread of resistant phenotypes of *M. tuberculosis* are nowadays a major health problem due to the reduced therapeutic options, high mortality rates and danger to the community if transmission of the bacillus is not readily stopped (World Health Organization, 2013).

Intrinsic resistance of *M. tuberculosis* to antimicrobial agents is mainly attributed to the reduced permeability of the cell wall due to the lipid-rich composition and the presence of mycolic acids that considerably decreases the intracellular access of antibiotics (Brennan and Nikaido, 1995). However, it cannot prevent completely their entrance. Other intrinsic mechanisms of resistance, such as efflux pumps, act synergistically with the permeability barrier to reduce the passage of antimicrobials across the bacterial cell wall (De Rossi et al., 2006; Piddock, 2006; Li and Nikaido, 2009). Efflux pumps usually confer low levels of drug resistance but play an important role in the evolution to high levels of resistance in *M. tuberculosis* (Machado et al., 2012). Prolonged exposure to subinhibitory concentrations of antituberculosis drugs facilitate the progressive acquisition of chromosomal mutations and provide the natural ground for the development of bacteria with high-level resistance phenotypes due to the acquisition of mutations in the antibiotic target. This chain of events is particularly relevant in long-term therapies such as that used in tuberculosis treatment, where a sustained pressure of sub-inhibitory concentrations of antibiotics can result in an increased efflux activity and allow the selection of spontaneous high-level drug resistant mutants (Machado et al., 2012; Schmalstieg et al., 2012).

A possible alternative to prevent the resistance generated by efflux is the chemical inhibition of these systems by molecules that act as inhibitors, the so called efflux inhibitors (EIs) that can act as treatment adjuvants to increase the activity of the antibiotics (Marquez, 2005). Such molecules are expected to reduce the intrinsic resistance of the bacteria by increasing the intracellular concentration of antibiotics even in highly resistant strains and reduce the frequency of emergence of resistant mutant strains (Mahamoud et al., 2007; Viveiros et al., 2010). The net result of blocking the efflux of an antimicrobial compound by the use of an EI is to decrease the threshold concentration (i.e., the minimum inhibitory concentration, MIC) of the antibiotic when the EI is used at concentrations devoid of any antibacterial activity. Many compounds have been reported as having inhibitory activity on mycobacterial efflux systems such as calcium channel blockers like verapamil, thioridazine, chlorpromazine, farnezol, reserpine, or uncouplers of the proton motive force such as carbonyl cyanide m-chlorophenyl hydrazone (CCCP) (Viveiros et al., 2012), but none has evolved toward clinical usage.

So far no MDR clinical strain was identified with high-level resistance attributed solely to overexpressed efflux pumps and the contribution of these systems to the overall level of resistance in MDR-TB clinical strains, irrespective of the existing mutations for drug targets in the bacteria, has not been thoroughly explored. In the present study we have explored the contribution of the efflux mechanisms to the overall resistance to isoniazid, rifampicin, amikacin and ofloxacin in five MDR (two of which pre-XDR) *M. tuberculosis* clinical isolates from Brazil by (i) the analysis of the synergistic effect of the EIs verapamil, thioridazine and chlorpromazine on the MICs of the antibiotics and ethidium bromide by a tetrazolium microplate-based assay (TEMA) on checkerboard format; (ii) the analysis of real-time efflux activity, using ethidium bromide as efflux substrate, by a semi-automated fluorometric method in presence and absence of each EI; and (iii) the analysis of mRNA transcriptional levels of selected efflux pump genes in these strains.

## Materials and methods

### *M. tuberculosis* strains, its characterization, and selection criteria

The *M. tuberculosis* strains included in this study were selected from the culture collection of the Mycobacteria Laboratory of Interdisciplinary Area of Biomedical Sciences, Núcleo de Pesquisa em Microbiologia Médica (NUPEMM), Faculty of Medicine, Federal University of Rio Grande, Brazil, and Fundação Estadual de Produção e Pesquisa em Saúde no Centro de Desenvolvimento Científico e Tecnológico. The strains were previously characterized using the proportion method on Löwenstein-Jensen for isoniazid, rifampicin, amikacin, and ofloxacin, and DNA sequencing to search for mutations associated with resistance (data not shown) (Maschmann et al., 2013). The strains to be studied were chosen based upon their resistance to isoniazid, rifampicin, amikacin and ofloxacin and presence of the most common mutations found in clinical isolates. The sampling comprises five multidrug resistant strains (three resistant to isoniazid and rifampicin; one resistant to isoniazid, rifampicin and amikacin; one resistant to isoniazid, rifampicin and ofloxacin—the two latest colloquially considered pre-XDR), whose phenotypic and genotypic characterization is shown in Table 1. The *M. tuberculosis* H37Rv ATCC27294 reference strain was used as control.

**Table 1:** **Phenotypic and genotypic characterization of the *M. tuberculosis* strains**.

**Strain**	**Phenotype**	**Genotype**				
		***kat*G**	***inh*A**	***rpo*B**	***gyr*A**	***rrs***
FURG-1	MDR	S315T	Wt	S531L	Wt	Wt
FURG-2	MDR	S315T	Wt	S531L	Wt	Wt
FURG-3	MDR	S315T	Wt	S531L	Wt	Wt
FURG-4	MDR	S315T	Wt	D516Y	A90V	Wt
FURG-5	MDR	D735A	Wt	S531L	Wt	A>G 1401
H37Rv	Susceptible	Wt	Wt	Wt	Wt	Wt

*MDR, multidrug resistant; R, resistant; Wt, wild-type*.

### Antimicrobials, efflux inhibitors, and ethidium bromide

Isoniazid, rifampicin, ofloxacin, amikacin, chlorpromazine, thioridazine, verapamil, ethidium bromide, glucose, phosphate buffered solution (PBS), and 3-4,5-dimethylthiazol-2-yl-2, 5-diphenyl tetrazolium bromide (MTT) were purchased from Sigma-Aldrich (St. Louis, MO, USA). Verapamil, chlorpromazine, thioridazine, isoniazid, amikacin, and ethidium bromide were prepared in sterile distilled water; rifampicin was prepared in dimethyl sulfoxide (DMSO), and ofloxacin in 1% hydroxide chloride in water. Stock solutions were stored at −20°C. The work solutions were prepared at the day of the experiments. The 10X MTT stock solution was prepared in ultrapure sterile water and used at 1:1 in 10% Tween 80 (v/v).

### Determination of minimum inhibitory concentrations (MIC)

For the determination of the MICs of the antibiotics, EIs, and the efflux substrate ethidium bromide, the strains were grown in Middlebrook 7H9 (DIFCO, Madrid, Spain) plus 10% OADC supplement (oleic acid/albumin/dextrose/catalase) (Becton and Dickinson, Diagnostic Systems, Sparks, MD, USA) at 37°C until they reached an OD_600_ of 0.8. Afterwards, the inoculum was prepared by diluting the bacterial cultures in 7H9/OADC medium to a final density of approximately 10^5^ cells/ml (Eliopoulos and Moellering, 1996). The MICs were determined by a TEMA (Caviedes et al., 2002) with slight modifications. Briefly, aliquots of 0.1 ml of inoculum were transferred to each well of the plate that contained 0.1 ml of each compound at concentrations prepared from two-fold serial dilutions in 7H9/OADC medium. The concentration ranges used are shown in Table S1 of the Supplementary data. Growth controls with no drug and a sterility control were included in each plate assay. Two hundred microliters of sterile deionized water was added to all outer-perimeter wells of the 96-well plates to reduce evaporation of the medium in the wells during the incubation. The inoculated plates were sealed in plastic bags and incubated at 37°C for 7 days. After this period, MTT was added to each well to a final concentration of 2.5% and the plates incubated overnight. The bacterial viability was registered for each well based on the MTT color change. Here, the amount of color generated is directly proportional to the number of viable cells and a precipitate of cells stained black can be observed in the bottom of the well. The MIC was defined as the lowest concentration of compound that totally inhibited bacterial growth (Gomez-Flores et al., 1995). All assays were carried out in triplicate.

### Determination of fractional inhibitory concentration (FIC) and modulation factor (MF)

Two-dimensional broth microdilution checkerboard assay (Eliopoulos and Moellering, 1996) combined with TEMA, was performed to assess the effect of the EIs verapamil, thioridazine and chlorpromazine in combination with isoniazid, rifampicin, amikacin and ofloxacin against the *M. tuberculosis* strains. Two hundred microliters of sterile deionized water was added to all outer-perimeter wells of the plates to prevent evaporation of the medium during the incubation. The plates were prepared by dispensing the serially diluted antibiotic in the x-axis and the inhibitors in the y-axis. The concentration range used for each compound is shown in Table S2. Aliquots of 0.1 ml of inoculum were transferred to each well of the 96-well plate and incubated at 37°C. In all assays were included growth and sterility controls. The inoculated plates were sealed in plastic bags and incubated at 37°C for 7 days and the results interpreted as described above. The modulation factor (MF) was used to quantify the effect of the inhibitors on the MIC of antibiotics and ethidium bromide (**Formula 1**) (Gröblacher et al., 2012). The modulation factor reflects the reduction of MIC values of a given antibiotic in the presence of the efflux inhibitor and was considered to be significant when MF ≥ 4 (four-fold reduction). The effect of each efflux inhibitor on the activity of each antibiotic was determined by means of fractional inhibitory concentration (FIC) determination according to **Formula 2**. The FIC was calculated only for the antibiotic as the concentration of the efflux inhibitor to be used in each combination corresponds to ≤¼ of its MIC, which is considered to be devoid of antibacterial activity. The FIC indexes were interpreted using the criteria established by Pillai et al. (2005). However, since we are only evaluating the individual FIC for the antibiotic and not the sum of the FICs, or FIC index (Σ*FIC*_antibiotic_), the results were interpreted as follows: FIC ≤ 0.25, synergism; FIC > 0.25 < 2, indifference and FIC ≥ 2, antagonism. As such, an individual FIC of ≤ 0.25, indicative of a four−fold reduction, was assumed as synergy. We considered FIC >$ 0.25 as indifferent activity due to the inherent variability of the method (Odds, 2003). The FIC for the combinations was classified as ND (non-determinable) when the MICs of the compounds alone were greater than the highest, less than, or equal to the lowest concentration tested (Moody, 1992). For combinations that showed tendencies for synergy, isobolograms were constructed, by plotting changes in the MIC of antibiotics as a function of the EIs concentration, using GraphPad Prism V5.01 software (La Jolla, USA). Synergy is illustrated by a concave isobol and antagonism, by a convex isobol. All assays were carried out in triplicate.

$$MF=\frac{MIC_{antiobiotic}}{MIC_{combination}}$$

**Formula 1. Modulation factor (MF) determination**. *MIC_antibiotic_* corresponds to the MIC of the antibiotic; *MIC_combination_* corresponds to the MIC of the antibiotic in the presence of efflux inhibitor.

$$FIC_{antibiotic}=\frac{MIC_{combination}}{MIC_{alone}}$$

**Formula 2. Fractional inhibitory concentration (FIC) determination**. *MIC_combination_* corresponds to the MIC determined in the presence of the antibiotic and the inhibitor; *MIC_alone_* corresponds to the MIC of the antibiotic alone.

### Evaluation of efflux activity by real-time fluorometry

The assessment of efflux activity on a real-time basis was performed using a semi-automated fluorometric method, as previously described (Viveiros et al., 2010; Machado et al., 2012). The *M. tuberculosis* strains were grown in 10 ml of 7H9/OADC with 0.05% Tween 80 at 37°C until they reach an OD_600_ of 0.8. For the accumulation assays, the cultures were centrifuged at 3500 rpm for 3 min, the supernatant discarded and the pellet washed in PBS. The OD_600_ was adjusted to 0.8 with PBS. In order to determine the concentration of ethidium bromide that establish the equilibrium between efflux and influx, aliquots of 0.05 ml of the bacterial suspension were added to 0.2 ml microtubes containing 0.05 ml different concentrations of ethidium bromide that ranged from 0.625 to 3 μg/ml with and without 0.4% glucose. The assays were conducted at 37°C in a Rotor-Gene 3000™ (Corbett Research, Sydney, Australia), using the 530 nm band-pass and the 585 nm high-pass filters as the excitation and detection wavelengths, respectively. Fluorescence data was acquired every 60 s for 60 min. The selected concentration of ethidium bromide was further used for the evaluation of the capacity of the EIs to retain ethidium bromide inside the cells. The EIs verapamil, thioridazine and chlorpromazine were tested at ½ MIC with and without 0.4% glucose and ethidium bromide at the equilibrium concentration determined for each strain and the assays performed like described above. The inhibitory activity of the compounds was determined by the calculation the relative final fluorescence (RFF) value according to **Formula 3** (Machado et al., 2011):

$$RFF=\;\frac{RF_{treated}-\;RF_{untreated}}{RF_{untreated}}$$

**Formula 3. Relative final fluorescence (RFF) determination**. *RF_treated_* corresponds to the relative fluorescence at the last time point of EtBr accumulation curve (minute 60) in the presence of an inhibitor; and the *RF_untreated_* corresponds to the relative fluorescence at the last time point of the EtBr accumulation curve of the untreated control tube. EtBr, ethidium bromide.

For the efflux assays, strains were exposed to conditions that promote maximum accumulation of ethidium bromide, i.e., ethidium bromide equilibrium concentration for each strain, no glucose, presence of the efflux inhibitor that caused maximum accumulation (in all cases verapamil), and incubation at 25°C for 1 h (Viveiros et al., 2010). Before incubation, the cultures were centrifuged at 3500 rpm for 3 min, resuspended in PBS, centrifuged again and OD_600_ adjusted to 0.4. The suspension was incubated with ethidium bromide and verapamil under the conditions described above. Aliquots of 0.05 ml of cells were transferred to 0.2 ml microtubes containing 0.05 ml of each efflux inhibitor at ½ MIC without ethidium bromide. Control tubes with only cells and cells with and without 0.4% glucose were included. Fluorescence was measured in the Rotor-Gene™ 3000 and data was acquired every 30 s for 30 min. Efflux activity was quantified by comparing the fluorescence data obtained under conditions that promote efflux (presence of glucose and absence of efflux inhibitor) with the data from the control in which the mycobacteria are under conditions of no efflux (presence of an inhibitor and no energy source). Thus, the relative fluorescence corresponds to the ratio of fluorescence that remains per unit of time, relatively to the ethidium bromide-loaded cells.

### RNA isolation, RT-qPCR, and quantification of efflux pump mRNA levels

Total RNA from *M. tuberculosis* cultures was extracted using the RNeasy Mini kit (QIAGEN, GmbH, Hilden, Germany) as previously described (Machado et al., 2012). The concentration and quality of total RNA was measured using a NanoDrop 1000 spectrophotometer (Thermo Scientific, Waltham, USA). The primers sequences used for the analysis of the efflux genes *mmpL7*, *mmr*, *Rv1258c*, *p55*, *Rv2469*, *efpA*, and the transcriptional regulator *whiB7* are described in Table 2. The RT-qPCR assay was performed in a Rotor-Gene™ 3000 thermocycler and followed the protocol recommended for use with the QuantiTect SYBR Green RT-PCR Kit (QIAGEN) with the following amplification program: reverse transcription for 30 min at 50°C; initial activation step for 15 min at 95°C; 35 cycles of denaturation at 94°C for 30 s, annealing at 52°C for 30 s and extension at 72°C for 30 s; a final extension step at 72°C for 5 min; and an additional step at 50°C for 15 s followed by melt analysis (50–99°C). The quantification of the relative mRNA levels of the genes in the strains exposed and non-exposed to subinhibitory concentrations of the antibiotics was performed using the standard curve method (Bustin, 2000). Standards consisted of known numbers of molecules prepared from cDNA PCR products of the target genes. The PCR products were purified by gel extraction (QIAquick Gel Extraction Kit, QIAGEN), quantified by spectrophotometry and the molecular weight determined. The number of molecules/μ l of the standards was calculated according to the following **Formula 4** (Yin et al., 2001):

$$molecules/\mu l=\frac{6.023\times 10^{23}\times C\times OD_{260}}{MW}$$

**Formula 4. Quantification of mRNA copy number**. In this formula, *C* corresponds to 50 ng/μ l for DNA and *MW*, to the molecular weight of the target gene.

**Table 2:** **Primers used in this study**.

**Gene**	**Primer Sequence (5′-3′)**	**Amplification product (bp)**	**References**
16S_Fw	CAA GGC TAA AAC TCA AAG GA	197	Rodrigues et al., 2012
16S_Rv	GGA CTT AAC CCA ACA TCT CA		
*mmpL7*_Fw	TAC CCA AGC TGG AAA CAA	214	
*mmpL7*_Rv	CCG TCA GAA TAG AGG AAC CAG		
*p55*_Fw	AGT GGG AAA TAA GCC AGT AA	198	
*p55*_Rv	TGG TTG ATG TCG AGC TGT		
*efpA*_Fw	ATG GTA ATG CCT GAC ATC C	131	
*efpA*_Rv	CTA CGG GAA ACC AAC AAA G		
*mmr*_Fw	AAC CAG CCT GCT CAA AAG	221	
*mmr*_Rv	CAA CCA CCT TCA TCA CAG A		
*Rv1258c_Fw*	AGT TAT AGA TCG GCT GGA TG	268	
*Rv1258c_Rv*	GTG CTG TTC CCG AAA TAC		
*Rv2459_Fw*	CAT CTT CAT GGT GTT CGT G	232	Machado et al., 2012
*Rv2459_Rv*	CGG TAG CAC ACA GAC AAT AG		
*whiB7_Fw*	TCG AGG TAG CCA AGA CAC T	109	Machado, 2014
*whiB7_Rv*	TCG AAT ATC TCA CCA CCC CA		

*Fw, forward; Rv, reverse*.

To generate the standard curve, each RNA sample was successively diluted in nuclease-free water, the *C_t_*-values of each dilution measured in triplicate, plotted against the logarithm of their initial template copy numbers and the *R*^2^ obtained to evaluate the performance of the assay. The PCR amplification efficiency was controlled by the slope of the standard curve. To compensate variations in the input RNA and efficiency of the reverse transcription step, the results were normalized against the *M. tuberculosis* 16S rDNA reference gene. Data is presented as the n-fold difference relative to the control (non-exposed condition or, when necessary, the reference strain) plus standard deviation (±SD).

## Results

### Evaluation of the synergistic effect between efflux inhibitors and antibiotics

The MICs of the antibiotics isoniazid, rifampicin, amikacin and ofloxacin, the efflux substrate ethidium bromide, as well as the MICs of the EIs verapamil, thioridazine, and chlorpromazine, for each of the strains enrolled in this study, are given in Table 3. The FICs and MF obtained for each combination are listed in Table 4. All the multidrug resistant strains (*n* = 5) presented high-level resistance to isoniazid (5/5) and high- (4/5) and intermediate-level resistance to rifampicin (1/5). High-level resistance to ofloxacin was observed in one multidrug resistant strain, FURG-4. Resistance to amikacin (high-level) was detected only in one multidrug resistant strain, FURG-5. A mutation associated with the resistant phenotype was identified in all five multidrug resistant strains (Table 1). The combination between the antibiotics and the EIs tested at ½ of its MIC consistently produced non-determinable FICs since the combination interaction was always bellow the concentration range tested (see Tables S1, S2). This data indicates that the effect of these compounds at ½ of MIC, as commonly tested in the literature and in previous works of our group (Viveiros et al., 2010), is due to its antibacterial effect and not due exclusively to the inhibition of efflux activity (Machado, 2014). As such, for a more accurate and precise analysis of the effect of the EIs on the MIC values of the antibiotics and efflux substrate, the results were compared at ¼ MIC of each efflux inhibitor, where all the FICs (for all the strains) are inside the concentration range tested (Tables 3, 4).

**Table 3:** **Minimum inhibitory concentrations of the antibiotics, efflux inhibitors, and ethidium bromide for the *M. tuberculosis* strains**.

**Strain**	**MIC (μg/ml)**							
	**INH**	**RIF**	**AMK**	**OFX**	**VP**	**TZ**	**CPZ**	**EtBr**
FURG-1	10	2048	2	1	512	15	15	8
FURG-2	10	1024	2	2	512	15	30	8
FURG-3	5	1024	1	1	512	15	15	4
FURG-4	5	16	2	16	512	15	30	4
FURG-5	512	1024	640	1	512	15	30	8
H37Rv	0.1	0.5	2	2	512	15	15	4

*INH, isoniazid; RIF, rifampicin; AMK, amikacin; OFX, ofloxacin; VP, verapamil; TZ, thioridazine; CPZ, chlorpromazine; EtBr, ethidium bromide*.

**Table 4:** **Synergistic activity of the efflux inhibitors in combination with antituberculosis drugs against the *M. tuberculosis* strains**.

**Antibiotics**	**EIs**	**Strains**																	
		**FURG-1**			**FURG-2**			**FURG-3**			**FURG-4**			**FURG-5**			**H37Rv**		
		**MIC (μg/ml)**	**FIC**	**MF**	**MIC (μg/ml)**	**FIC**	**MF**	**MIC (μg/ml)**	**FIC**	**MF**	**MIC (μg/ml)**	**FIC**	**MF**	**MIC (μg/ml)**	**FIC**	**MF**	**MIC (μg/ml)**	**FIC**	**MF**
**INH**	**No EI**	**10**	-	-	**10**	-	-	**5**	-	-	**5**	-	-	**512**	**-**	**-**	**0.1**	**-**	**-**
	+VP ¼	3	0.3	3	3	0.3	3	3	0.6	2	5	1	1	512	1	1	**0.025**	**0.25**	**4**
	+TZ ¼	3	0.3	3	3	0.3	3	3	0.6	2	5	1	1	512	1	1	**0.025**	**0.25**	**4**
	+CPZ ¼	3	0.3	3	3	0.3	3	3	0.6	2	**1.25**	**0.25**	**4**	512	1	1	**0.025**	**0.25**	**4**
**RIF**	**No EI**	**2048**	-	-	**1024**	**-**	**-**	**1024**	-	-	**16**	-	-	**1024**	**-**	**-**	**0.5**	**-**	**-**
	+VP ¼	**512**	**0.25**	**4**	**256**	**0.25**	**4**	**16**	**0.015**	**64**	**0.5**	**0.03**	**32**	**128**	**0.125**	**8**	**0.125**	**0.25**	**4**
	+TZ ¼	2048	1	1	512	0.5	2	**32**	**0.03**	**32**	**1**	**0.06**	**16**	1024	1	1	0.25	0.5	2
	+CPZ ¼	2048	1	1	512	0.5	2	512	0.5	2	**0.5**	**0.03**	**32**	1024	1	1	0.25	0.5	2
**OFX**	**No EI**	**1**	**-**	**-**	**2**	-	-	**1**	-	-	**16**	-	-	**1**	-	-	**2**	-	-
	+VP ¼	0.5	0.5	2	**0.5**	**0.25**	**4**	0.5	0.5	2	16	1	1	0.5	0.5	2	1	0.5	2
	+TZ ¼	1	1	1	**0.5**	**0.25**	**4**	0.5	0.5	2	16	1	1	0.5	0.5	2	1	0.5	2
	+CPZ ¼	0.5	0.5	2	1	0.5	2	0.5	0.5	2	16	1	1	0.5	0.5	2	1	0.5	2
**AMK**	**No EI**	**2**	-	-	**2**	**-**	**-**	**1**	**-**	**-**	**2**	**-**	**-**	**640**	**-**	**-**	**2**	**-**	**-**
	+VP ¼	**0.5**	**0.25**	**4**	**0.5**	**0.25**	**4**	**0.25**	**0.25**	**4**	**0.5**	**0.25**	**4**	640	1	1	**0.5**	**0.125**	**4**
	+TZ ¼	1	0.5	2	**0.5**	**0.25**	**4**	**0.25**	**0.25**	**4**	**0.5**	**0.25**	**4**	640	1	1	1	0.5	2
	+CPZ ¼	1	0.5	2	**0.25**	**0.125**	**4**	0.5	0.5	2	**0.5**	**0.25**	**4**	**160**	**0.25**	**4**	**0.5**	**0.125**	**4**
**EtBr**	**No EI**	**8**	**-**	**-**	**8**	-	-	**4**	-	-	**4**	-	-	**8**	-	-	4	-	-
	+VP ¼	**1**	**0.125**	**8**	**0.5**	**0.06**	**16**	**0.25**	**0.0625**	**16**	**1**	**0.25**	**4**	**0.5**	**0.06**	**4**	**0.5**	**0.125**	**8**
	+TZ ¼	**2**	**0.25**	**4**	**2**	**0.25**	**4**	**0.5**	**0.125**	**8**	2	0.5	2	**1**	**0.125**	**8**	2	0.5	2
	+CPZ ¼	8	1	1	8	1	1	4	1	1	**1**	**0.25**	**4**	**2**	**0.25**	**4**	4	1	1

*MIC, Minimum inhibitory concentration; FIC, fractional inhibitory concentration; MF, modulation factor; INH, isoniazid; RIF, rifampicin; AMK, amikacin; OFX, ofloxacin; VP, verapamil; TZ, thioridazine; CPZ, chlorpromazine; EtBr, ethidium bromide; EI, efflux inhibitor. Values in bold type indicate synergistic interactions*.

**Figure 1:**
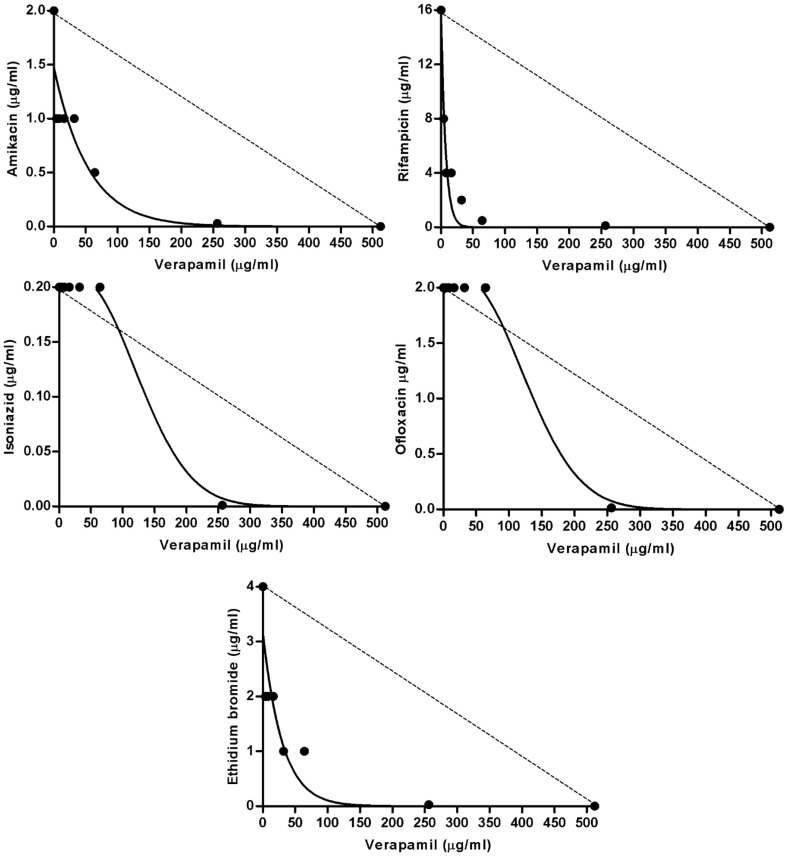
**Synergic effect of the combination of amikacin, rifampicin, isoniazid, ofloxacin and ethidium bromide, plus verapamil**. In the figure is represented the isobolograms for the *M. tuberculosis* strain FURG-4 as an example. The dashed line represents the hypothetical indifferent effect. The concave isobol observed represents the synergic effect.

As can be seen in Table 4, the MIC values of isoniazid were reduced (MF ≥ 3) in 2/5 multidrug resistant strains (FURG-1 and -2) by the three inhibitors, and in 1/5 only by chlorpromazine (FURG-4). For this last strain, the FIC result indicated a synergist effect. For strain FURG-5, none of the inhibitors was able to reduce the high-level resistance to isoniazid (MIC of 512 μg/ml). For H37Rv, the MIC for isoniazid was reduced by all the inhibitors originating a FIC of 0.25 indicative of synergism. Concerning rifampicin resistance, the MIC values were reduced with verapamil in all rifampicin resistant strains by four-fold (2/5), eight-fold (1/5), 32-fold (1/5), and 64-fold (1/5). Thioridazine was able to reduce the MIC of rifampicin in 2/5 strains and chlorpromazine in 1/5. Of note, strain FURG-4, which displays intermediate level of resistance to rifampicin and harbors a rare mutation on codon 516, was the strain for which the resistance to rifampicin was reduced in the presence of the three inhibitors to levels similar of that of the susceptible H37Rv reference strain (Table 4). The MIC for rifampicin of the susceptible H37Rv reference strain was reduced four-fold only by verapamil. Resistance to amikacin was found in strain FURG-5 to which the MIC was reduced four-fold with chlorpromazine. For the amikacin susceptible strains the MIC values were reduced by two- to four-fold depending on the strain. Resistance to ofloxacin was reduced only in strain FURG-2 by four-fold with verapamil and thioridazine. Concerning ethidium bromide, the MIC values were reduced in all strains with verapamil by four- to 16-fold, and with thioridazine and chlorpromazine by four- to eight-fold in 4/6 and 2/6 strains, respectively. The FICs determined for the individual synergistic interactions described above ranged from 0.25 (MF = 4) to 0.015 (MF = 64) (Table 4) and is illustrated in Figure 1 for the four antibiotics and ethidium bromide plus verapamil against *M. tuberculosis* FURG-4 as an example for the remaining strains tested. Noteworthy, no antagonistic interaction was observed in this study.

### Assessment of efflux activity by real-time fluorometry

**Figure 2:**
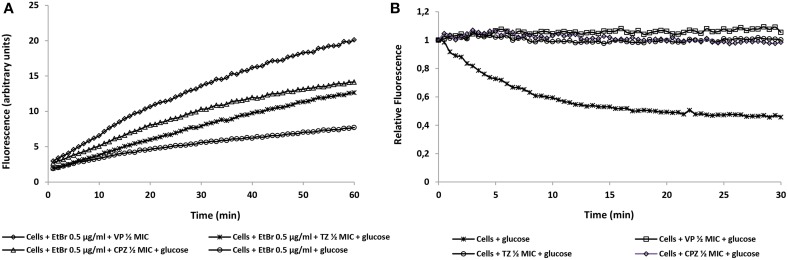
**Effect of the efflux inhibitors on the accumulation (A) and efflux (B) of ethidium bromide at 0.5 μg/ml by *M. tuberculosis* FURG-5, as an example**. Assays were performed at 37°C in the presence of glucose. Concentrations of verapamil (VP), thioridazine (TZ), chlorpromazine (CPZ) are at ½ their MIC (Table 3). EtBr, ethidium bromide.

After determining the synergistic effect between the inhibitors and antibiotics, the inhibitors were evaluated for their ability to inhibit efflux activity by the *M. tuberculosis* strains in study. Real-time fluorometry was applied to study their behavior in the presence of the broad efflux substrate ethidium bromide with and without glucose as the source of metabolic energy. First, was determined the lowest concentration that causes minimal accumulation of ethidium bromide, i.e., the concentration for which there is an equilibrium between influx and efflux of the substrate. The lowest concentration that resulted in equilibrium between the influx and efflux of ethidium bromide was 0.25 μg/ml for the reference strain H37Rv, 0.5 μg/ml for strains FURG-2 to FURG-5, and 1 μg/ml for strain FURG-1. These results clearly indicate that the clinical drug resistant *M. tuberculosis* strains can handle higher concentrations of ethidium bromide than the reference strain, which is suggestive of the presence of more active efflux systems in these strains. After this, the EIs under study were tested under the equilibrium concentration of ethidium bromide identified for each strain. In Figure 2A, we can observe the effect of the EIs on the accumulation of ethidium bromide by *M. tuberculosis* FURG-5, in the presence of glucose, as an example. Due to the large number of graphs describing the effects of verapamil, thioridazine and chlorpromazine, with and without glucose, on the efflux activity of the other five *M. tuberculosis* strains studied, the RFF indexes were calculated and the results presented in tabular form (Table 5). The index of activity of the three EIs tested against the *M. tuberculosis* strains was calculated with the aid of the **Formula 3** (see Material and Methods). The RFF index is a measure of how effective the compound is on the inhibition of ethidium bromide efflux (at a given non-inhibitory concentration) by comparison of the final fluorescence at the last time point (60 min) of the treated cells with the cells treated only with ethidium bromide. The inhibitor that presents the highest ethidium bromide accumulation rate was verapamil, followed by thioridazine and chlorpromazine, as previously demonstrated (Machado et al., 2012; Rodrigues et al., 2012). The results showed that all strains studied presented with efflux activity which can be inhibited in the presence of the EIs (Figure 2B as an example of the results obtained with all strains tested). Efflux activity was more pronounced in the clinical strain FURG-5 (Figure 2B); strains FURG-1 to - 4 presented significant and similar EtBr efflux activities but less than FURG-5, whilst H37Rv presented a basal efflux activity, assessed by the semi-automated fluorometric method (data not shown). Moreover, it was also noticed that the accumulation of ethidium bromide by the *M. tuberculosis* strains is not affected by the presence of glucose (Table 5), revealing that the external energization of *M. tuberculosis* cells is not determinant to guarantee an optimal active efflux, as previously noticed (Machado et al., 2012; Rodrigues et al., 2012). These results provide further evidence that efflux activity is an intrinsic characteristic of susceptible and drug resistant *M. tuberculosis* strains and is directly involved in antibiotic resistance in the *M. tuberculosis* clinical strains, irrespectively of the presence or absence of mutations that confer antibiotic resistance.

**Table 5:** **Relative final fluorescence (RFF) based on accumulation of EtBr**.

**Strains**	**RFF of the efflux inhibitors**					
	**Verapamil**		**Thioridazine**		**Chlorpromazine**	
	**− glucose**	**+ glucose**	**− glucose**	**+ glucose**	**− glucose**	**+ glucose**
FURG-1	0.82	0.47	0.66	0.29	0.32	0.01
FURG-2	**1.36**	**1.01**	0.63	0.33	0.39	0.21
FURG-3	0.71	0.53	0.21	0.27	0.07	−0.04
FURG-4	**1.58**	**1.22**	0.76	0.45	0.64	0.46
FURG-5	**1.62**	**1.48**	**1.37**	**1.54**	**1.06**	**1.01**
H37Rv	**1.57**	**2.27**	0.77	**1.31**	0.52	0.88

*Accumulation of EtBr at 0.25 μg/ml (H37Rv), 0.5 μg/ml (FURG-2 to 5), and 1 μg/ml (FURG-1), in the presence of glucose at 0.4%. The most efficient inhibitors are in bold type*.

### Quantification of efflux pump mRNA by RT-qPCR

**Figure 3:**
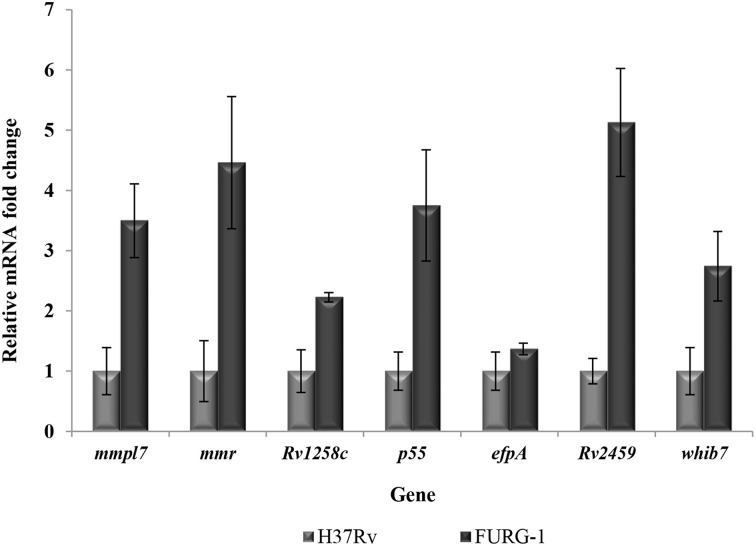
**Quantification of the mRNA transcriptional levels (n-fold) for the efflux pump genes in strain FURG-1**. The mRNA levels of the genes *mmpl7*, *mmr*, *p55*, *Rv1258c*, *Rv2459*, *efpA* and the transcriptional regulator *whib7* were compared with the H37Rv reference strain.

To further analyze the contribution of efflux pumps to antibiotic resistance in these strains, six efflux pump genes, *mmpl7*, *mmr*, *p55*, *Rv1258c*, *Rv2459* and *efpA*, and the transcriptional regulator *whib7*, were selected to examine changes in mRNA transcriptional levels (Table 2). The quantification of efflux pump mRNA level of the *M. tuberculosis* clinical isolates is shown in Table 6. When comparing the mRNA levels of each efflux pump gene for each isolate with that of the H37Rv reference strain, no increase in the expression of these genes was detected in these strains, with the exception of strain FURG-1. This strain presents a two- to five-fold increase in mRNA levels for all genes tested (Figure 3). To analyze if the presence of an antibiotic could induce an increase in mRNA transcriptional levels, the strains were exposed to ½ of the respective MIC of the antibiotic to which the strains was shown to be resistant and the transcription levels determined against the non-exposed counterpart. The results are presented in Table 6. Increased mRNA levels were found in strains FURG-2 for all seven genes upon exposure to rifampicin, and six genes for isoniazid (Table 6). Strain FURG-4 demonstrates increased mRNA levels for all genes tested upon exposure to ofloxacin. Strain FURG-5 shows only a marginal increase in the expression of the *efpA* gene. Concerning strains FURG-1, FURG-3, FURG-4, and FURG-5 it was noticed a general decrease in the transcript numbers of all efflux pump genes after the exposure to isoniazid, rifampicin, or amikacin.

**Table 6:** **Relative quantification of efflux pump gene mRNA quantity in the *M. tuberculosis* strains exposed to antibiotics**.

**Strain**	**Condition**	**Relative mRNA gene quantity (±SD) of:**						
		***mmpl7***	***mmr***	***Rv1258c***	***p55***	***efpA***	***Rv2459***	***whib7***
	**Non-exposed**	**1.0**	**1.0**	**1.0**	**1.0**	**1.0**	**1.0**	**1.0**
**FURG-1**								
	+INH	0.01 ± 0.00	0.05 ± 0.01	0.02 ± 0.00	0.01 ± 0.00	0.07 ± 0.00	0.01 ± 0.00	0.02 ± 0.00
	+RIF	0.02 ± 0.00	0.04 ± 0.01	0.01 ± 0.00	0.03 ± 0.00	0.01 ± 0.00	0.01 ± 0.00	0.01 ± 0.00
**FURG-2**								
	+INH	**94.56 ± 1.64**	**137.21 ± 0.00**	**20.09 ± 1.05**	**24.65 ± 2.56**	**10.41 ± 3.16**	0.41 ± 0.39	**2.35 ± 0.16**
	+RIF	**172.05 ± 10.42**	**249.20 ± 12.95**	**90.18 ± 15.08**	**84.90 ± 5.14**	**56.80 ± 2.95**	**16.59 ± 4.19**	**13.08 ± 1.13**
**FURG-3**								
	+INH	0.23 ± 0.03	0.16 ± 0.02	0.03 ± 0.01	0.18 ± 0.04	0.80 ± 0.29	0.13 ± 0.01	0.04 ± 0.00
	+RIF	0.02 ± 0.00	0.05 ± 0.00	0.01 ± 0.00	0.06 ± 0.03	0.06 ± 0.04	0.05 ± 0.00	0.06 ± 0.03
**FURG-4**								
	+INH	0.28 ± 0.05	0.17 ± 0.01	0.22 ± 0.01	0.53 ± 0.11	0.76 ± 0.03	0.37 ± 0.03	0.42 ± 0.06
	+RIF	0.01 ± 0.00	0.03 ± 0.00	0.05 ± 0.00	0.01 ± 0.00	0.02 ± 0.00	0.01 ± 0.00	0.01 ± 0.00
	+OFX	**4.45 ± 0.15**	**2.41 ± 0.25**	**2.75 ± 0.29**	**4.17 ± 0.29**	**4.02 ± 0.14**	**2.96 ± 0.51**	**5.51 ± 0.38**
**FURG-5**								
	+INH	1.00 ± 0.10	0.60 ± 0.24	0.10 ± 0.06	0.76 ± 0.05	**1.15 ± 0.04**	0.48 ± 0.03	0.20 ± 0.01
	+RIF	0.07 ± 0.04	0.01 ± 0.01	0.01 ± 0.00	0.01 ± 0.01	0.02 ± 0.00	0.02 ± 0.00	0.01 ± 0.00
	+AMK	0.01 ± 0.00	0.08 ± 0.02	0.02 ± 0.00	0.01 ± 0.00	0.01 ± 0.00	0.01 ± 0.00	0.01 ± 0.00

*INH, isoniazid; RIF, rifampicin; OFX, ofloxacin; AMK, amikacin. Data is presented as the n-fold difference relative to the control (non-exposed condition) plus standard deviation (±SD). Values in bold type were considered to be overexpression*.

## Discussion

Understanding the mechanism by which *M. tuberculosis* develops resistance and how this can be reduced is essential to shorten and improve tuberculosis treatment. Mutations in the genes related with antibiotic resistance and efflux of drugs have been shown to contribute to the development of drug resistance in *M. tuberculosis* (Louw et al., 2011; Silva and Palomino, 2011; Machado et al., 2012). In this work, we aimed first to determine whether the combination of antituberculosis drugs and putative EIs act synergistically against a panel of drug resistant *M. tuberculosis* strains, and second to study the contribution of efflux systems to the overall drug resistance in these strains.

We applied a two-dimensional checkerboard procedure combined with a TEMA to evaluate the interactions of first and second line drugs and the efflux substrate ethidium bromide with the EIs verapamil, thioridazine, and chlorpromazine against a panel MDR *M. tuberculosis* clinical strains. The results showed a synergistic interaction between all antituberculosis drugs and the EIs although with different degrees of synergism (Figure 1 as an example and Table 4). The interaction between rifampicin, amikacin or ethidium bromide plus the inhibitors was significant even at low concentrations of the inhibitors, whereas, for isoniazid and ofloxacin the synergic interaction was observed only at higher concentrations of the inhibitors. Significant reduction of the MIC values of ofloxacin was achieved only in one susceptible strain and no reduction was observed in the ofloxacin-resistant strain. This result indicates that, although fluoroquinolones are considered to be common substrates efflux pumps in other bacterial pathogens (Costa et al., 2011), efflux activity seems to have little contribution for ofloxacin resistance in multidrug resistant *M. tuberculosis* strains, as previously shown (Machado, 2014). Resistance to isoniazid was only moderately reduced, as these isoniazid resistant strains harbor mutations within the *katG* gene (Table 1) associated with high-level resistance (Böttger, 2011; Cambau et al., 2015). Strain FURG-5 presents a rare mutation in the *katG* gene, D735A (Wei et al., 2003), and the highest level of isoniazid resistance. Although rifampicin is not assumed to be a common substrate of efflux pumps, it was possible to reduce rifampicin MICs for all mutant strains by at least one of the EIs. Although we cannot assume reversal of resistance due to the absence of breakpoints for this methodology, the rifampicin MIC values for strain FURG-4 reached values similar to the fully susceptible reference strain H37Rv, despite the presence of a mutation. The mutation D516Y in *rpoB* gene has been described by others as been associated with different levels of susceptibility, from sensitive to high-level resistance (Williams et al., 1998; Somoskovi et al., 2006; Cambau et al., 2015). Resistance to amikacin was reduced by at least one of the inhibitors in drug susceptible strains. Concerning the amikacin-resistant strain, the resistance was reduced by four-fold with chlorpromazine. Kigondu et al. (2014) have recently reported that combinations between chlorpromazine and aminoglycosides results in synergic activity against *Mycobacterium smegmatis*. In this study, verapamil was the efflux inhibitor that demonstrated stronger activity against these strains which are in accordance with previous reports (Rodrigues et al., 2009, 2011, 2012; Louw et al., 2011; Machado et al., 2012; Gupta et al., 2013). Moreover, the results demonstrated that these EIs are active against both antibiotic-susceptible and -resistant strains indicating that the effect of these compounds is not dependent of the mutational profile of the strain. Additionally, it is likely these inhibitors have a wide range of activity acting on several efflux systems instead of been specific of a particular efflux pump.

In order to correlate the data obtained by the MICs determination and efflux activity, we applied a semi-automated fluorometric method using ethidium bromide as an indicator of efflux activity. The accumulation of increasing concentrations of ethidium bromide by the *M. tuberculosis* clinical strains, when comparing with the pan-susceptible H37Rv strain, clearly demonstrated that the clinical drug resistant strains, except FURG-3, possess enhanced efflux activity which could be inhibited in the presence of the EIs verapamil, thioridazine and only marginally by chlorpromazine (Table 5). We have recently observed the same kind of results with multi- and extensively drug resistant *M. tuberculosis* strains (Machado, 2014).

For the analysis of efflux pump gene expression, we first compared the mRNA levels of the clinical strains with the antibiotic susceptible reference strain, *M. tuberculosis* H37Rv. We observed an increase in the mRNA levels of all efflux genes tested in strain FURG-1 when compared with the reference strain. Regarding the remaining strains no efflux pump gene was found to be overexpressed using the H37Rv strain as a reference. As such, the clinical strains were exposed to ½ of the MIC of the antibiotics and the corresponding mRNA levels were compared with those of the same strain grown in a drug-free condition. We noticed an increase in the mRNA levels of almost all genes in strains FURG-2 exposed to isoniazid and rifampicin and FURG-4 exposed to ofloxacin. We also observed that the strains expressing efflux pumps shown the expression of the whole panel of genes tested and not a particular gene, as previously shown (Machado et al., 2012; Rodrigues et al., 2012; Machado, 2014). These results showed that these clinical strains are prepared to expel toxic compounds possibly as a consequence of the constant pressure to which they were subjected in the clinical setting (Costa et al., 2011; Machado et al., 2012). We did not notice a straight correlation between the efflux pump gene expression and the reduction of the antibiotic resistance levels by the EIs. Although the effect demonstrated by the EIs on the resistance levels of the antibiotics combined with the results obtained with the real-time fluorometry clearly supports the involvement of efflux pumps in the overall resistance in these strains, it does not necessarily indicate efflux pump over expression. All strains presented high-level resistance to the antibiotics tested (Table 3) due to the presence of a mutation conferring resistance (Table 1) coupled with a component of efflux demonstrated by the reduction of the resistance levels with the inhibitors (Table 4). The extent of the later will depend on the resistance level conferred by the mutation plus the different environmental pressures to which the strain was exposed. It is expected that clinical strains do not need to increase the amount of efflux pumps in the membrane to survive when exposed to subinhibitory concentrations of the antibiotics to which they are resistant. Additionally, we notice that some genes were downregulated upon exposure to the antibiotics (Table 6). At the present we do not know the reason, although we can hypothesize that (i) gene downregulation occurs due to an antibiotic killing effect, or (ii) the downregulation of these efflux systems occurs at the expense of the overproduction of other unknown efflux systems or (iii) gene downregulation can be due to the presence of mutations in the genes encoding the efflux pumps. Further experiments would be necessary to test these hypotheses.

Tuberculosis continues to be a deadly disease worldwide and new approaches for its treatment are necessary. The identification of clinical isolates of *M. tuberculosis* that presented an efflux component as part of their resistant phenotype has a major impact in tuberculosis therapeutics as well in the development of new drugs. With the increase of multi- and extensively drug resistant strains, there are few alternatives available to treat these patients. The data presented evidences a possible therapeutic value for compounds that have the ability to inhibit mycobacterial efflux pumps via the retention of co-administered antimycobacterial drugs that are subject to efflux (Viveiros et al., 2012). The rationale and procedures used in this study proved to be useful to evaluate the presence of active efflux systems in drug resistant *M. tuberculosis* strains. In our hands, they afford quantitative and reproducible results and proved to be an inexpensive, rapid, high-throughput assay to predict the activity of EIs against drug susceptible and resistant strains of *M. tuberculosis*. In conclusion, this study strengths the notion that intrinsic efflux activity also contributes to the overall resistance in drug resistant clinical isolates of *M. tuberculosis* bearing the most frequent mutations for resistance and that the inhibition of this efflux activity by compounds such as thioridazine and verapamil can promote the clinical effect of the antibiotics that are subject to efflux, highlighting the urgent need of animal studies to guide the future progress of these compounds into combinational clinical trials (Viveiros et al., 2012; Gupta et al., 2013; Dutta and Karakousis, 2014).

### Conflict of interest statement

The authors declare that the research was conducted in the absence of any commercial or financial relationships that could be construed as a potential conflict of interest.
