# Supplementary material for: Enhancement of antibiotic activity by efflux inhibitors against multidrug resistant Mycobacterium tuberculosis clinical isolates from Brazil
Source: Front Microbiol. 2015 Apr 28;6:330. doi: 10.3389/fmicb.2015.00330 (PMC4412083; doi:10.3389/fmicb.2015.00330)
Supplement: Supplementary file 1 [file Table1.DOCX]

**Enhancement of antibiotic activity by efflux inhibitors against multidrug resistant Mycobacterium tuberculosis clinical isolates from Brazil**

# Supplementary material

**Table S1: Concentrations used for MIC determination.**

| **Strains** | **Concentrations (µg/ml)** | | | | | | | |
| --- | --- | --- | --- | --- | --- | --- | --- | --- |
|  | **INH** | **RIF** | **OFX** | **AMK** | **VP** | **TZ** | **CPZ** | **EtBr** |
| **FURG-1** | 0.08-10 | 8-2048 | 0.015-2 | 0.015-2 | 2-512 | 0.46-30 | 0.93-60 | 0.125-16 |
| **FURG-2** | 0.08-10 | 8-1024 | 0.015-2 | 0.015-2 | 2-512 | 0.46-30 | 0.93-60 | 0.125-16 |
| **FURG-3** | 0.312- 40 | 8-1024 | 0.015-2 | 0.015-2 | 2-512 | 0.46-30 | 0.93-60 | 0.125-16 |
| **FURG-4** | 0.312- 40 | 8-1024 | 0.015-2 | 0.015-2 | 2-512 | 0.46-30 | 0.93-60 | 0.125-16 |
| **FURG-5** | 0.08-256 | 8-1024 | 0.015-2 | 5-640 | 2-512 | 0.46-30 | 0.93-60 | 0.125-16 |
| **H37Rv** | 0.003-0.4 | 0.015-2 | 0.015-2 | 0.015-2 | 2-512 | 0.46-30 | 0.93-60 | 0.125-16 |

INH, isoniazid; RIF, rifampicin, OFX, ofloxacin; AMK, amikacin; VP, verapamil; TZ, thioridazine; CPZ, chlorpromazine; EtBr, ethidium bromide.
