# Supplementary material for: Enhancement of antibiotic activity by efflux inhibitors against multidrug resistant Mycobacterium tuberculosis clinical isolates from Brazil
Source: Front Microbiol. 2015 Apr 28;6:330. doi: 10.3389/fmicb.2015.00330 (PMC4412083; doi:10.3389/fmicb.2015.00330)
Supplement: Supplementary file 2 [file Table2.DOCX]

**Enhancement of antibiotic activity by efflux inhibitors against multidrug resistant Mycobacterium tuberculosis clinical isolates from Brazil**

# Supplementary material

**Table S2. Concentrations of the antibiotics, efflux inhibitors and EtBr used in the checkerboard assays.**

| **Strains** | **Concentrations (µg/ml)** | | | | | | | |
| --- | --- | --- | --- | --- | --- | --- | --- | --- |
|  | “x” axis | | | | | “y” axis | | |
|  | **INH** | **RIF** | **OFX** | **AMK** | **EtBr** | **VP** | **TZ** | **CPZ** |
| **FURG-1** | 0.025-10 | 0.5-2048 | 0.015-2 | 0.015-2 | 0.125-16 | 16-512 | 0.43-15 | 0.43-15 |
| **FURG-2** | 0.025-10 | 0.5-1024 | 0.015-2 | 0.015-2 | 0.125-16 | 16-512 | 0.43-15 | 0.93-30 |
| **FURG-3** | 0.05-20 | 0.5-1024 | 0.015-2 | 0.015-2 | 0.125-16 | 16-512 | 0.43-15 | 0.93-30 |
| **FURG-4** | 0.039-5 | 0.5-1024 | 0.015-2 | 0.015-2 | 0.125-16 | 16-512 | 0.43-15 | 0.93-30 |
| **FURG-5** | 0.025-10 | 0.5-1024 | 0.015-2 | 5-640 | 0.125-16 | 16-512 | 0.43-15 | 0.93-30 |
| **H37Rv** | 0.006-0.8 | 0.015-2 | 0.015-2 | 0.015-2 | 0.125-16 | 16-512 | 0.93-30 | 1.87-60 |

INH, isoniazid; RIF, rifampicin, OFX, ofloxacin; AMK, amikacin; VP, verapamil; TZ, thioridazine; CPZ, chlorpromazine; EtBr, ethidium bromide.
